# An American Text-Book of Obstetrics

**Published:** 1896-12

**Authors:** 


**An American Text-Book of Obstetrics.** RICHARD C. NORRIS, M.D., Editor. ROBERT L. DICKINSON, M.D., Art Editor. Two volumes. Pp. 1—492, 493—1009. London: THE REBMAN PUBLISHING COMPANY, LIMITED. 1896.

This truly magnificent work is introduced to us by the simple statement in its preface, that "Although our standard text-books of obstetrics have occasionally been *revised*, an entirely new text-book containing the writings of more than one individual has not appeared during the last decade." The two volumes before us are the joint work of fifteen eminent American obstetric physicians, who, with scarcely an exception, are men of ripe experience, and who have, moreover, the power of imparting the fruits of their experience to others.

Before we proceed to review in detail the different portions of this work, it is but just and right to make some preliminary remarks upon its general "getting up." This reflects the greatest credit upon the publishers. Evidently no expense has been spared, and the work has been done in the very best manner, equally as regards quality of paper, clearness of type, and the high artistic merit of the numerous engravings, some of which are beautifully coloured. As regards illustrations, there is indeed quite an *embarras de richesse*. Many of these are original, being drawn from nature or from photographs, whilst many others are delicate line engravings, copied on a small scale from the bold, life-sized designs of many of the old masters.

The arrangement of the different parts of this important "text-book" is exceedingly good and judicious. The first part begins with a very clear and complete description of the anatomy and physiology of the female generative organs. This part is profusely illustrated; but there is one picture in particular which forms the frontispiece of the work and is beautifully coloured. It represents a mesial section of the pelvis and its contained organs, and is admirably adapted to give a student a correct idea of the relations of these parts to one another. This section has been very properly confided to Dr. Piersol, Professor of Anatomy in the University of Pennsylvania. It contains a most complete description of every detail connected with the anatomy of the pelvic bones and the contained soft parts, and occupies no less than fifty-seven pages, nearly every one of which is beautifully illustrated. The same remark also applies to the physiology of pregnancy: in this he has received the valuable help of Dr. Palmer, Obstetrician and Gynæcologist to the Cincinnati Hospital, to whose experience and skill we owe the very judicious remarks on the changes induced in the maternal organism during pregnancy. Their section, however, on the diagnosis of pregnancy, treats of this subject rather too shortly, when we consider its immense importance, in actual practice, both to the young obstetric practitioner and to his patient. It is sometimes difficult to say which may be most damaged by a wrong diagnosis, the reputation of a young doctor or the mental and bodily condition of his patient. Yet in so complete and so elaborate a work as Norris's *Text-Book of Obstetrics* we find that only twenty pages both of letterpress and illustrations are devoted to this important subject. The deficiency here is most serious in the matter of illustrations. The same holds good with most of the symptoms and signs of pregnancy, but with two striking exceptions: in one, our knowledge of the changes produced by pregnancy in the breasts is derived almost entirely from the sense of sight; in the other (perhaps the most important of all the signs of pregnancy), viz. auscultation, from the sense of hearing. It is therefore obvious that exact drawings from nature of the size and colour of the areola are all-important for teaching purposes; but that as regards auscultation, illustrations of any kind are all but useless. The only way to learn it is by practice at the bedside. In Montgomery's classical work on the signs of pregnancy his singularly full and correct description of the changes in the breast is accompanied by no less than eight beautiful full-page drawings representing the nipple and areola in each progressive change which takes place in every month from the third to the ninth inclusive. From Montgomery's full and lucid description, illustrated by these beautiful plates, a student may easily learn all that is necessary for him to know on this subject. But in the "text-book" before us, instead of eight drawings, each in a separate page, they are all squeezed into one page; and to do this comfortably they are reduced to two-thirds of the natural size, and, greatest defect of all, they are not even coloured, although very well engraved. A plate of this kind deprives the student of more than half the information he might otherwise obtain from it. In each page of a volume the size of this one it would be easy to insert two drawings of life-size and colour. In a book so abounding in excellent work, like the one before us, we are sorry to have to point out so serious a deficiency; but we trust, by so doing, to lead to some alteration in a second edition.

The hygiene and management of pregnancy are also dealt with by Dr. Palmer, who, in a small space, gives much good advice as to clothing, diet, air and exercise in pregnancy. He recommends occasional examination of the urine, about once a month, because "albuminuria is present in at least from 5 to 10 per cent. of the cases of pregnant women;" and when this is so, there is danger of eclampsia. Sexual intercourse he discourages, especially during the earlier and later months.

The section on the pathology of pregnancy is a very long one, comprising 133 pages. No less than five authors have contributed to this, which, if we may judge from the reputation of each, illustrates the old proverb that "in the multitude of counsellors there is wisdom." For instance, Dr. Davis, of Philadelphia, writes a long and very complete description of various disorders, both general and local, during pregnancy, and their appropriate treatment, and also of acute infections, and accidents and surgical operations during this period; Dr. Etheridge, of Chicago, on diseases of the ovum and abortion, and the relations of one to the other; Dr. Kelly, of Baltimore, on that dangerous *lusus naturæ*, extra-uterine pregnancy; and lastly, Dr. Earle, of Chicago, whose article, on account of his death, was finished by Dr. Mergler, on that very interesting but very little known or understood subject, diseases of the fœtus *in utero*.

After a thorough description of pregnancy in all its bearings, we come, in the ordinary course of events, to the consideration of natural labour. This includes, in about twenty pages, a good description of the symptoms accompanying the first, second, and third stages of labour, by Dr. Dickinson, Obstetrician to Long Island College Hospital, New York, and is illustrated by some diagrams showing sections of the os uteri and pelvic floor during the process of dilatation and expulsion. These are very well done, and very useful in a practical point of view to students. The conduct of normal labour is described in a very clear and practical manner by Dr. Jewett, who is also Obstetrician to Long Island College Hospital. Like most American doctors, he attaches the greatest importance to antisepsis in labour, and gives minute and particular directions for enforcing it that might be found tedious to a practitioner who is suddenly called to a rapid case. Anæsthesia, for which he prefers chloroform, he uses but sparingly and prudently. He also describes the management of the three stages of labour, especially how to manage abdominal palpation, and examinations, external and internal. There are no less than nine plates, evidently from photographs, to show the mode of palpation. The utility of such plates is perhaps doubtful, for the knowledge of these manual operations has to be learned rather by the *tactus eruditus* at the bedside than by the sense of vision.

It is a gratifying proof of the confidence of his colleagues in Dr. Reynolds, of Boston and Harvard University, that so important a subject as the mechanism of labour should have been entrusted solely to him, and their confidence in him has not been misplaced. The mechanism of ordinary vertex presentations is very fully described and illustrated by a great number of most useful diagrams. The more untoward presentations of the head are also fully gone into, and the management of each variety is carefully described. Less attention, however, has been paid to presentations of the breech, inferior and superior extremities or cross births, and what are called compound presentations or prolapsed extremities. In cases of arrested breech we have found the fillet safer as regards the child, and more effectual than any other instrument. The worst kind of instrument is the forceps. If applied as in Figs. 277 and 278, it would either slip off or make injurious pressure on the child's viscera. We have quite given up applying forceps to the breech.

We have next to notice the second volume. It has much more to do with pathology than the first, and consequently with obstetric operations of every kind. It is exceedingly practical and excellent in every way, and is undoubtedly the more important of the two volumes. It begins with dystocia, and it is, perhaps, the best illustrated section of the book. The greater portion of this is written by Dr. Hirst, Professor of Obstetrics, University of Pennsylvania, to whom has been entrusted the subject of "Anomalies in the Forces of Labor." There are a large number of beautiful plates to show every kind of deformity which may cause pelvic contraction; and lastly, a chapter on dystocia caused by disease or monstrous growth of the fœtus, with quite a picture-gallery of teratology, showing specimens of every kind of single or double monstrosity of the most uncouth and repulsive description, Dr. Parvin, of Jefferson Medical College, Philadelphia, writes on accidents to the cord. In prolapsed cord he recommends reposition; but if that fails, podalic version to save the life of the child, if not already lost from pressure. Dystocia due to hemorrhage is the joint work of Dr. Parvin and Dr. Schwarz of St. Louis. It includes the three kinds mentioned by Rigby, viz., accidental, unavoidable, and

*post-partum.* Of these, the second, viz., unavoidable hemorrhage arising from placenta prævia, is usually considered the most dangerous. Of this the authors give a very full and judicious account. Accidental hemorrhage, however, which according to our experience is quite as dangerous, does not, we think, receive quite sufficient attention. The same remark does not apply to the treatment of *post-partum* hemorrhage, almost every well-known and well-established mode of treating this being mentioned. Nor does it apply to dystocia due to accidents, such as rupture of the uterus and inversion, and diseases of the mother, especially eclampsia, about which Dr. Parvin writes a long and very good account. In treating these cases, he justly recognises the value of occasional bleeding, as our fathers did, but thinks they were "wrong in making venesection the common remedy in eclampsia, but their sons are equally wrong in entirely rejecting it." He also thinks highly of hypodermic injections of tincture of veratrum viride, from ten to twenty minims; and treats of dystocia arising from a variety of other diseases.

The next section deals with the puerperium. The parts treating of physiology, its diagnosis, and management are written by Dr. Jewett. He gives a good and clear description of the process of involution of the uterus, by which that organ very nearly regains its former bulk, and this is well illustrated by four diagrams. The largest and most important part of this section is that which embraces the pathology of the puerperium. For the greater part of this, which is all very well done, Dr. Norris, the Lecturer on Clinical and Operative Obstetrics, University of Pennsylvania, is responsible. In the first portion, which treats of the injuries of the genital organs following labour, Dr. Schwarz is associated with him, and Dr. Norris writes on hæmatoma only. Dr. Garrigues, Professor of Obstetrics in the New York Post-Graduate School and Hospital, follows next with a long and learned dissertation on puerperal infection, including a valuable essay on antiseptic precautions, proving by figures the truth of the old adage that "Prevention is better than cure." Subinvolution is the origin of a series of diseased conditions to which Dr. Norris with much ability traces them, and describes and treats accordingly. He states that "subinvolution is the starting-point of numerous intrapelvic disorders," and that "usually in from six to ten weeks the physiological changes known as *involution* have been completed." Delay in this process, he says, is generally due to the retention of decomposed clots or other *débris*, and should be treated accordingly. To this cause he attributes secondary hemorrhages, uterine displacements, &c. He then contributes a series of very useful articles on anomalies of the nipples and breasts, diseases of the breasts, and anomalies of the milk-secretion. These are followed by diseases of the non-sexual organs which are due to other causes than puerperal infection; and lastly, by a short but very well-written and impressive article on rapid or sudden death in the puerperium, which is alike remarkable for the beauty of its style, its brevity, and the amount of information which it contains.

We are indebted for the next section to Dr. Etheridge and Dr. Earle, who treat of a little subject which is generally too much neglected in obstetric works, viz., the new-born infant. But yet it is one of great importance. In nine cases out of ten we find, in England, that the accoucheur who attends the mother is the one whom she consults if anything goes wrong with the baby. It begins with a short but very good and complete treatise, by Dr. Etheridge, on the physiology of the new-born infant, and contains some very interesting facts with regard to the gradual development of its perceptive faculties. He also makes some useful remarks on the management of premature infants, and the importance of maintaining their proper temperature by an apparatus like Auvard's incubator. Dr. Earle is responsible, however, for the greater and more important part of this section. He enters very fully into an account of both the major and minor ailments of infants and their treatment, and has done this duty most satisfactorily.

Dr. J. C. Cameron, of Montreal, writes on instrumental operations, with the exception of symphysiotomy, for which Dr. Jewett is responsible. Dr. Dickinson gives a good account of operations requiring the hands only, such as external, bipolar, and internal version. Dr. Hirst who deals with "Celiotomy for Sepsis in the Child-bearing Period," provides us with an admirable essay on a subject which requires great care in its management. All three parts of the section on obstetric surgery are very rich in illustrations, altogether as well executed as those which pervade both volumes.

An excellent index of thirty-three pages concludes this highly meritorious work, which is a *chef d'œuvre* of which the fifteen authors may be justly proud.

